# Hay-Wells syndrome: the challenges of a nine-year follow-up^☆^

**DOI:** 10.1016/j.abd.2023.09.012

**Published:** 2024-08-06

**Authors:** Rebecca Perez de Amorim, Maria Vitória Yuka Messias Nakata, Vitória Gabrielle De Paula Gonçalves, Ivanka Miranda de Castro, Gabriela Roncada Haddad, Luciana Patrícia Fernandes Abbade

**Affiliations:** Department of Infectology, Dermatology, Imaging Diagnosis and Radiotherapy, Faculty of Medicine, Universidade Estadual Paulista, Botucatu, SP, Brazil

Dear Editor,

The present report discusses the evolution and management of a case of Hay-Wells Syndrome (HWS), previously published in this journal, of a male patient, currently nine years old, followed since he was four months old.^1^ The patient was born at term by vaginal delivery to non-consanguineous parents and has healthy siblings. From birth he has shown ulcerated lesions on the trunk, scalp, face and upper limbs (Fig. 1); onychodystrophy (Fig. 2); ankyloblepharon filiforme adnatum – corrected in the delivery room; cleft palate; pinna deformity; and micropenis.^1^ He was hospitalized during the first three months of life due to transepidermal losses with water and electrolyte imbalances, skin infections, otitis, and pneumonia. He was initially referred with a diagnosis of epidermolysis bullosa; however, the various clinical findings allowed the diagnosis of HWS.

**Figure 1:**
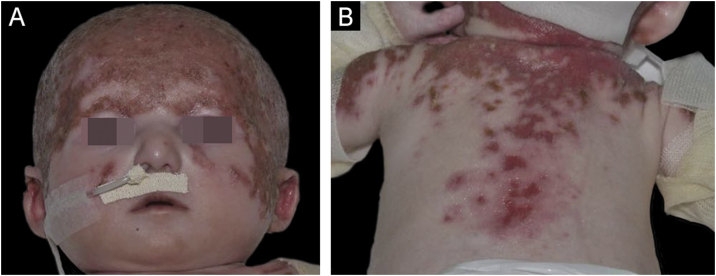
Child at four months of age showing in (A) exulcerations on the scalp and face and (B) exulcerations on the back and proximal region of the upper limbs.

**Figure 2:**
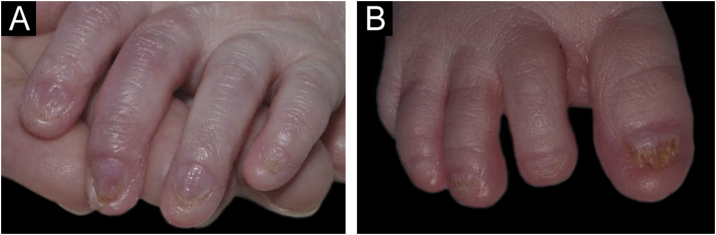
Nail changes in the newborn. Presence of onychodystrophy in all fingers (A) and all toes (B).

Since birth, the patient developed an intolerance and refused to accept an oral diet, requiring a nasoenteral tube, which was replaced by a gastrostomy tube at the age of three, when he also underwent correction of the cleft palate. At the age of four he was diagnosed with phimosis and balanopreputial adhesions, which have already been corrected, and glans hypospadias without the need for intervention. He started to show improvement regarding his tolerance to oral feeding only at seven years of age.

The skin exulcerations, especially those on the scalp, showed several episodes of exuberant granulation tissue and bacterial colonization since birth, without improvement in the first years of life (Fig. 3), despite local care and topical and systemic antimicrobial therapy. Several cultures of the exudate from these lesions were performed with positive results for community-associated methicillin-resistant *Staphylococcus aureus* (CA-MRSA) and *Pseudomonas aeruginosa*.

**Figure 3:**
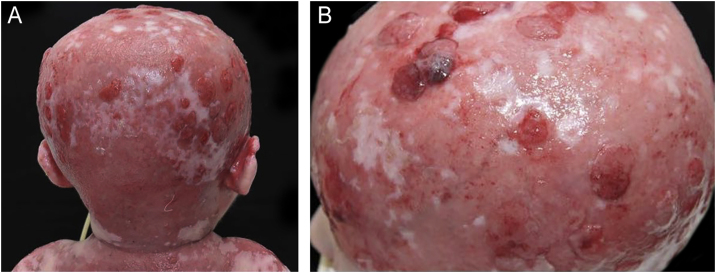
Child at one year old. Foci of exuberant granulation tissue and bacterial colonization seen in the parietal-occipital region of the scalp (A) and the parietal region (B).

The initial skin care was focused on the gentle removal of desquamation and crusts and the use of non-adherent dressings. For the exuberant granulation tissue, several sessions of superficial surgical debridement were performed with topical anesthesia and the use of specific dressings that absorbed the exudate, preferably with silver, to control and resolve local bacterial colonization. Initially, foam dressings with silver (hydropolymers) were chosen, as they can be cut into the required shape, have good exudate absorption power, and control moisture loss through evaporation, maintaining thermal insulation and a humid environment, with changes every four days. Aiming to improve patient quality of life, alleviate pain, accelerate healing and prevent episodes of colonization and infection.

Since the age of seven, the patient has not shown new exulcerations and no longer needs specific treatments. During the current dermatological examination, the pinna deformity, micropenis, alopecia, madarosis and onychodystrophy were identified, in addition to hypodontia, conical teeth, hypo/hyperchromic scars, mainly on the face, cervical region, scalp and anterior trunk; and hypertrophic scars on the upper limbs, anterior trunk and back (Fig. 4). The patient reported generalized anhidrosis and heat intolerance, evidenced on examination as generalized xerosis, without desquamation.

**Figure 4:**
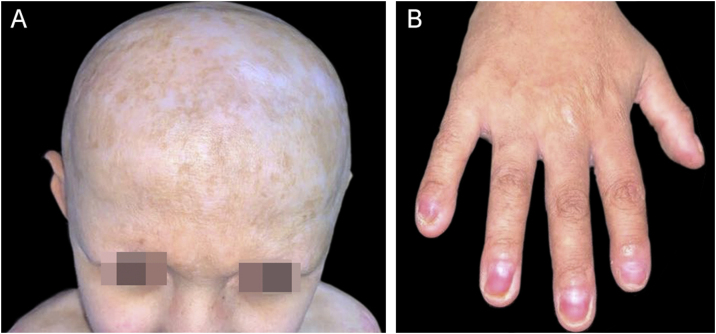
Child at nine years old, showing (A) cicatricial alopecia and (B) onychodystrophy in all fingers besides hyponychia in the 1st and 5th fingers.

He is currently undergoing follow-up with neuropediatrics (mild psychomotor neurodevelopment delay), speech therapy (speech difficulty), gastropediatrics, urology, otorhinolaryngology (punctate perforation of the tympanic membrane due to recurrent otitis), ophthalmology (using artificial tears), nutrition (short stature) and dermatology.

Several autosomal dominant syndromes result from mutations in the p63 gene, such as EEC (Ectrodactyly; Ectodermal dysplasia; Clefting) syndrome, ADULT (Acro-Dermato-Ungual-Lacrimal-Tooth) syndrome, limb-mammary syndrome, SHFM (Split-Hand/Foot Malformation) syndrome and HWS or AEC (Ankyloblepharon-ectodermal defects-cleft lip/palate) syndrome. These syndromes include ectodermal dysplasia, limb changes, and orofacial clefts. The different phenotypes are justified by different mutations that can occur in the same gene, either with gain or loss of function. The p63 gene encodes six different proteins that will have their functional effects altered in different ways, depending on the syndrome.^2^, ^3^, ^4^, ^5^, ^6^ Approximately 30% of the patients have an affected parent, while 70% of the cases arise from a *de novo* mutation.^5^

HWS/AEC syndrome shares some common features with EEC (Ectrodactyly, Ectodermal dysplasia, and facial clefting) syndrome. However, ectodermal involvement is much more severe in HWS/AEC syndrome. In the latter, scalp injuries occur more frequently and are more severe. Moreover, the facial cleft, when present in the EEC syndrome, is always a lip cleft (with or without a palate cleft), while only a cleft palate occurs in HWS. Limb involvement in the AEC syndrome is minimal or absent.^6^

The exulcerations in HWS are common at birth, with the risk of leading to cicatricial alopecia and hypotrichosis. Around 70% of newborns have ankyloblepharon filiforme adnatum with varying degrees of severity. There may also be agenesis or atresia of the lacrimal points, leading to conjunctivitis or chronic blepharitis. Most patients have nail dystrophy and dental abnormalities, such as hypodontia and conical teeth. Sweating can be reduced, generating thermal intolerance. A palate cleft with or without a lip cleft occurs in all cases. Changes in tongue position and speech difficulties have also been described.^5^, ^7^, ^8^, ^9^

In HWS, the necessary intensive care and subsequent individualized monitoring must be guaranteed according to the demands displayed throughout childhood development and adulthood, requiring continued multidisciplinary care. In fact, after the period of early childhood – when the risk of severe complications such as infections and fluid loss is greater – the overall prognosis is good, with normal life expectancy, as evidenced in this case, with significant improvement in skin lesions after the age of seven.^5^, ^9^

## Financial support

None declared.

## Authors’ contributions

Rebecca Perez de Amorim: Design and planning of the study; drafting and editing of the manuscript; collection, analysis and interpretation of data; intellectual participation in the propaedeutic and/or therapeutic conduct of the studied cases; critical review of the literature; critical review of the manuscript; approval of the final version of the manuscript.

Maria Vitória Yuka Messias Nakata: Drafting and editing of the manuscript; collection, analysis and interpretation of data; critical review of the literature; critical review of the manuscript.

Vitória Gabrielle De Paula Gonçalves: Drafting and editing of the manuscript; collection, analysis and interpretation of data; critical review of the literature; critical review of the manuscript.

Ivanka Miranda de Castro: Collection, analysis and interpretation of data; intellectual participation in the propaedeutic and/or therapeutic conduct of the studied cases.

Gabriela Roncada Haddad: Design and planning of the study; effective participation in research orientation; critical review of the literature; critical review of the manuscript; approval of the final version of the manuscript.

Luciana Patrícia Fernandes Abbade: Design and planning of the study; drafting and editing of the manuscript; collection, analysis and interpretation of data; intellectual participation in the propaedeutic and/or therapeutic conduct of the studied cases; critical review of the literature; critical review of the manuscript; approval of the final version of the manuscript.

## Conflicts of interest

None declared.

## References

[1] Tonolli V.M., Stolf H.O., Tonello C.S., Pires R.B., Abbade L.P. (2014). Syndrome in question. Hay-Wells syndrome. An Bras Dermatol.

[2] Fete M., vanBokhoven H., Clements S.E., McKeon F., Roop D.R., Koster M.I. (2009). International research symposium on ankyloblepharon-ectodermal defects-cleft lip/palate (AEC) syndrome. Am J Med Genet A.

[3] Bertola D.R., Kim C.A., Albano L.M., Scheffer H., Meijer R, van Bokhoven H. (2004). Molecular evidence that AEC syndrome and Rapp-Hodgkin syndrome are variable expression of a single genetic disorder. Clin Genet.

[4] Koster M.I. (2010). p63 in skin development and ectodermal dysplasias. J Invest Dermatol.

[5] Serra G., Antona V., Giuffré M., Li Pomi F., Lo Scalzo L., Piro E. (2021). Novel missense mutation of the TP63 gene in a newborn with Hay-Wells/Ankyloblepharon-Ectodermal defects-Cleft lip/palate (AEC) syndrome: clinical report and follow-up. Ital J Pediatr.

[6] Brunner H.G., Hamel B.C.J., van Bokhoven H. (2002). The p63 gene in EEC and other syndromes. J Med Genet.

[7] Julapalli M.R., Scher R.K., Sybert V.P., Siegfried E.C., Bree A.F. (2022). Dermatologic findings of ankyloblepharon-ectodermal defects-cleft lip/palate (AEC) syndrome. Am J Med Genet A.

[8] Dishop M.K., Bree A.F., Hicks M.J. (2009). Pathologic changes of skin and hair in ankyloblepharon-ectodermal defects-cleft lip/palate (AEC) syndrome. Am J Med Genet A.

[9] Rosa DJ de F., Machado R.F., Martins Neto M.P., Sá A.A., Gamonal A. (2010). Hay-Wells syndrome: a case report. An Bras Dermatol.

